# A problem at any age: a case report of congenital malrotation with bowel ischemia in an 84-year-old

**DOI:** 10.1186/s12893-022-01482-6

**Published:** 2022-01-29

**Authors:** Marisa E. Schwab, Sage P. Kramer, Aya Bashi, Taehyun P. Chung, Claudia M. Mueller

**Affiliations:** 1 Department of Surgery, University of California San Francisco, 505 Parnassus Ave, San Francisco, CA 94143 USA; 2 Department of Surgery, California Pacific Medical Center, San Francisco, CA USA; 3 Department of Radiology, California Pacific Medical Center, San Francisco, CA USA; 4 Geisel School of Medicine at Dartmouth, Hanover, NH USA; 5 Division of Pediatric Surgery, Stanford University School of Medicine, Stanford, CA USA

**Keywords:** Malrotation, Elderly, Ladd’s, Paraduodenal hernia, Case report

## Abstract

**Background:**

Malrotation with bowel ischemia is classically thought of as a disease of infants. However, the true prevalence of malrotation in both the pediatric and adult population is unknown due to the unclear number of asymptomatic patients.

**Case presentation:**

A previously healthy 84-year-old man with no prior abdominal surgeries presented with an acute abdomen and was found on CT to have small bowel located in the right hemiabdomen and an abnormal SMA-SMV relationship suggestive of intestinal malrotation, as well as pneumatosis intestinalis. He underwent an exploratory laparotomy, where he was found to have a paraduodenal space which did not contain any bowel but was the likely source of an internal hernia. His duodenojejunal flexure was located to the right of the spinal column, the cecum in the left lower quadrant, a thick congenital band at the proximal jejunum, and multiple Ladd’s bands at the level of the duodenum. The bowel appeared viable and a Ladd’s procedure was performed. The patient had an uneventful post-operative course.

**Conclusions:**

There is a lack of guidelines regarding screening for and management of asymptomatic malrotation in older children and adults. However, the traditional thinking is that asymptomatic malrotation diagnosed after two years of age poses minimal risk. This case illustrates the potential risk of an internal hernia in the setting of malrotation at any time during one’s lifetime.

**Supplementary Information:**

The online version contains supplementary material available at 10.1186/s12893-022-01482-6.

## Background

Intestinal malrotation is a congenital anomaly that occurs secondary to the failure of normal intestinal rotation with retroperitoneal fixation of the midgut during embryogenesis [1]. Malrotation is uncommon, with an estimated prevalence of 0.17% [2]. It is classically thought that 90% of patients with malrotation present in the first year of life with the majority (64–80%) presenting in the first month [3]. The management of older children with asymptomatic or incidental malrotation remains controversial. The 2015 American Pediatric Surgical Association (APSA) outcomes and evidence-based practice committee recommended observation for older children with asymptomatic malrotation [4]. Here, we report a case of malrotation and paraduodenal hernia causing bowel ischemia in an elderly patient.

## Case presentation

**Fig. 1:**
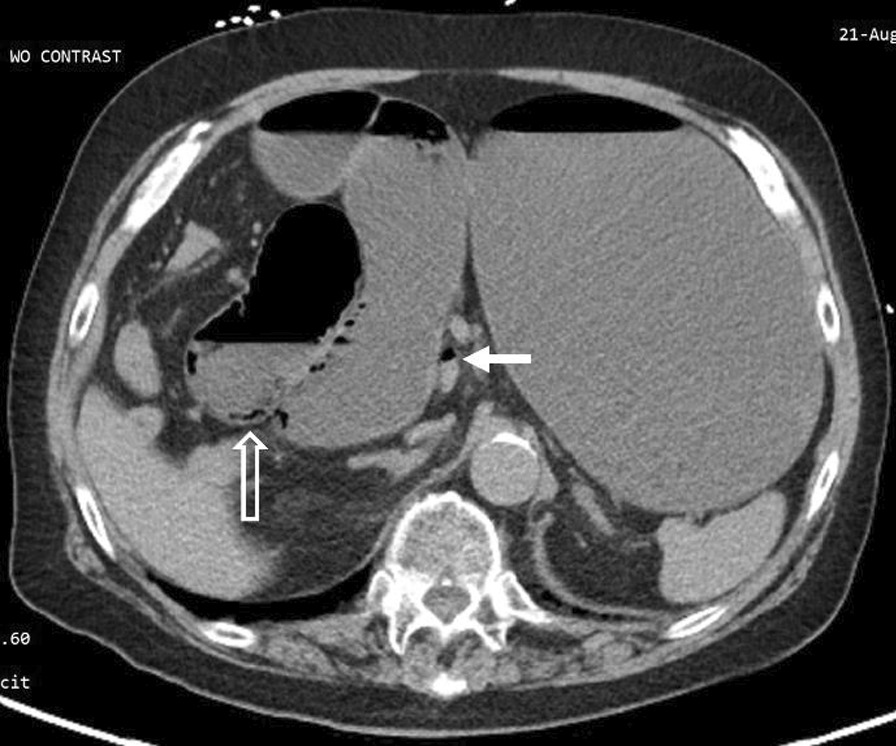
Pneumatosis intestinalis. CT of the abdomen without contrast. Gas and fluid distension of the stomach and duodenum. Mild duodenal pneumatosis intestinalis (hollow arrow). Gas in the main portal vein (solid arrow)

**Fig. 2:**
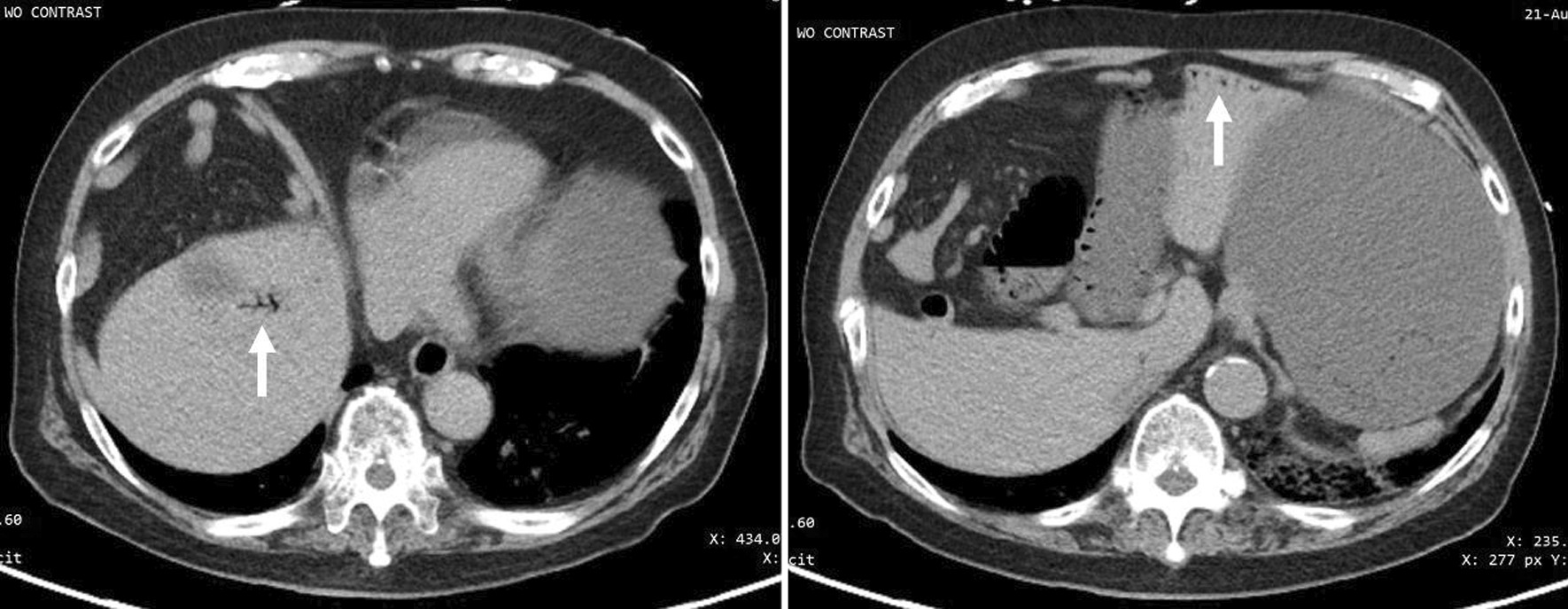
Portal venous gas. CT of the abdomen without contrast. Branching lucencies in the peripheral anti-dependent liver (solid arrows) compatible with portal venous gas

An 84-year-old healthy man with no prior abdominal surgeries and whose past medical history included only atrial fibrillation and hypertension presented to the emergency department due to 3 days of diffuse abdominal pain and persistent emesis with inability to tolerate any liquids or solids. He denied any longstanding symptoms of weight loss, abdominal pain, or nausea. He was noted to have a lactate of 10.2 mmol/L, a leukocytosis to 21.3 K/uL, and a creatinine of 3.07 mg/dL. A CT showed significant distension of the stomach, duodenum, and proximal jejunum up to 4.8 cm with a transition point in the right upper quadrant, with bowel ischemia evidenced by duodenal pneumatosis intestinalis (Fig. 1) and portal venous gas (Fig. 2.)

**Fig. 3:**
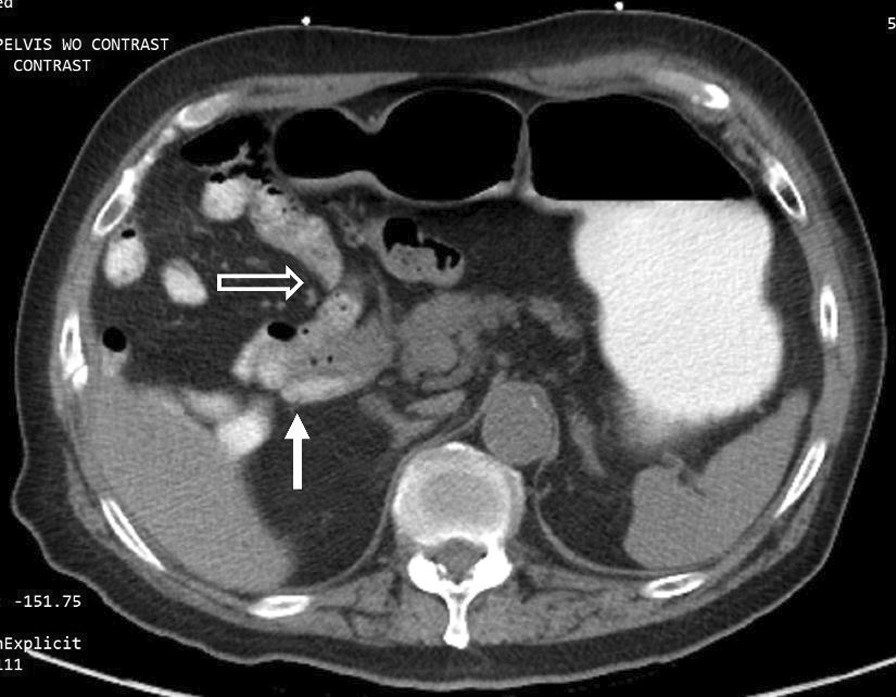
Location of D3. CT of the abdomen without intravenous contrast and with positive oral contrast. The duodenum (hollow arrow) fails to pass under the superior mesenteric artery to the left upper quadrant. The duodenojejunal flexure (solid arrow) is abnormally located in the right upper quadrant

**Fig. 4:**
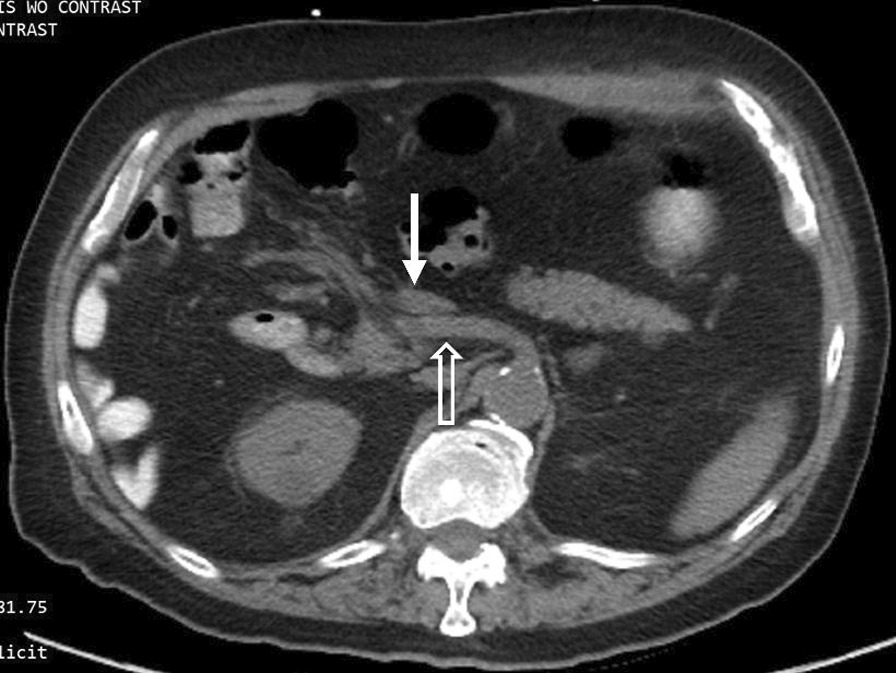
SMA-SMV relationship. CT of the abdomen without intravenous contrast and with positive oral contrast. The superior mesenteric artery (hollow arrow) is abnormally positioned to the right of the superior mesenteric vein (solid arrow) near the mesenteric root

**Fig. 5:**
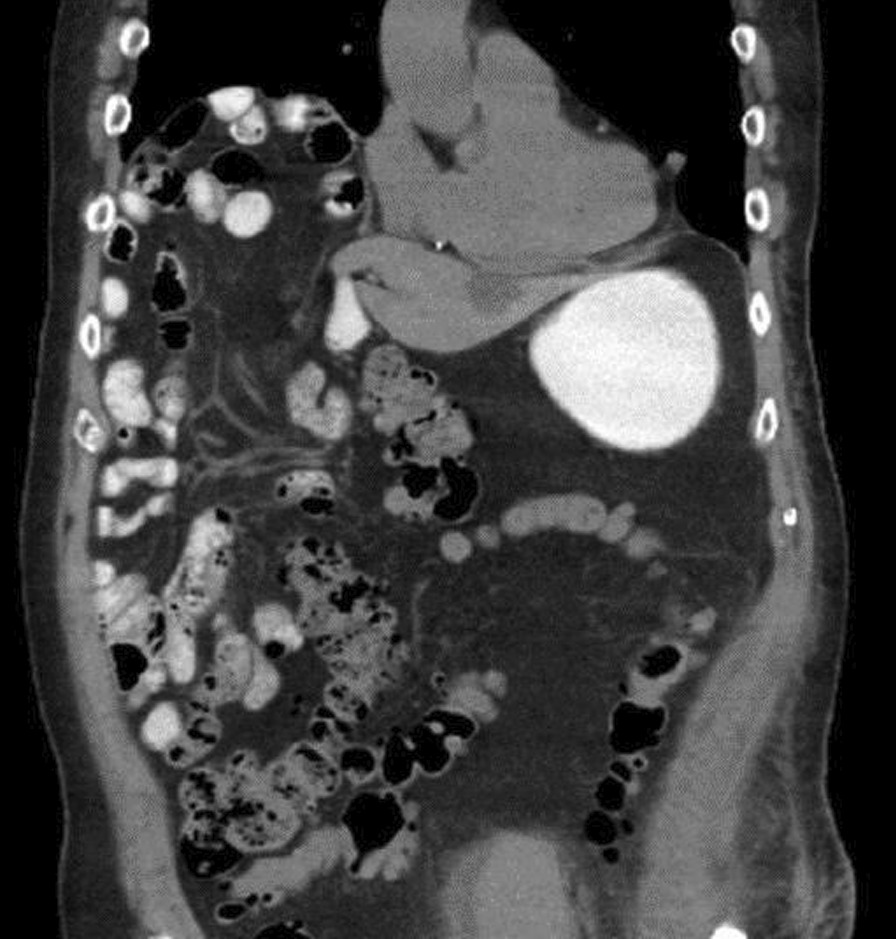
Location of proximal small bowel. CT of the abdomen and pelvis without intravenous contrast and with positive oral contrast. The entire small bowel is abnormally located in the right hemiabdomen and the colon predominantly to the left of the small bowel

**Fig. 6:**
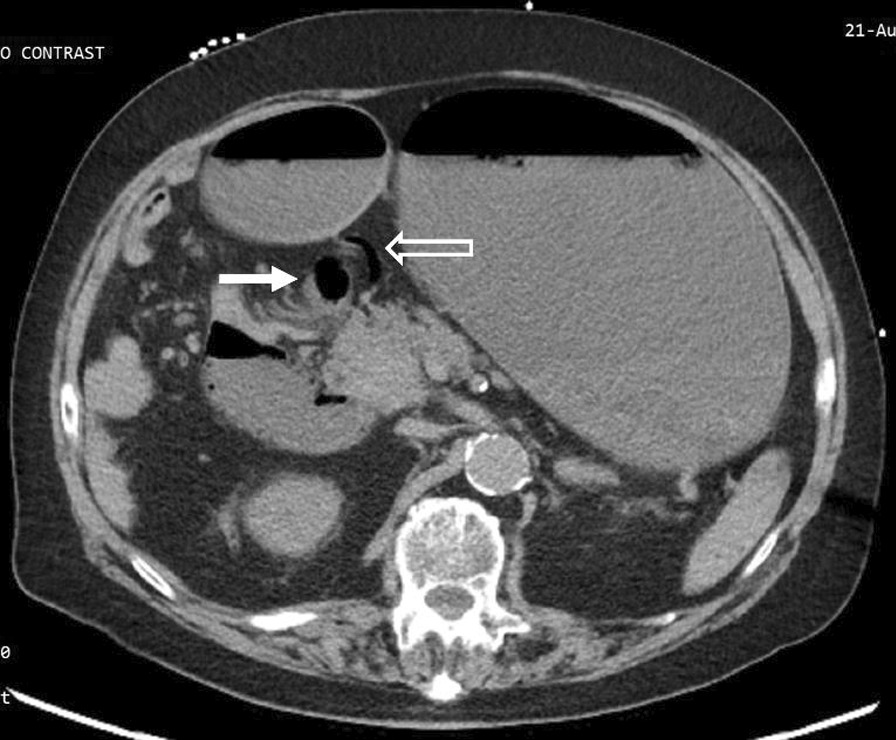
Right paraduodenal hernia. CT of the abdomen without contrast. Transition point (solid arrow) at the third segment of the duodenum, posterior to the ascending mesocolon in the expected location of the fossa of Landzert. Suggestive of a right paraduodenal hernia. Incidentally noted gas in a superior mesenteric vein tributary (hollow arrow)

The duodenojejunal flexure was abnormally located in the right upper quadrant, and the duodenum failed to pass under the superior mesenteric artery to the left upper quadrant (Fig. 3). There was an abnormal superior mesenteric artery (SMA) and superior mesenteric vein (SMV) relationship (Fig. 4), and the entire small bowel was in the right hemiabdomen (Fig. 5), suggestive of malrotation. His CT was compared to one four years prior, performed when the patient presented with abdominal ecchymosis after a fall, and at the time malrotation was not reported yet was clearly present. The constellation of findings on the current CT was suspicious for a strangulated right paraduodenal hernia (Fig. 6).

On exam, the patient’s abdomen was distended but soft with minimal diffuse tenderness. A nasogastric tube drained 3 L of dark bilious output, and the patient was taken emergently to the operating room for an exploratory laparotomy. There was a thick congenital band at the level of the proximal jejunum, with decompressed distal small bowel (Additional file 1: Fig. S1). The duodenojejunal flexure was located to the right of the spinal column. There was a paraduodenal space that did not contain any bowel but that was likely the location of a right paraduodenal internal hernia. There were several bands at the level of the duodenum that were divided, allowing the duodenum to be straightened out. While the small bowel was injected and edematous, it appeared viable. The cecum was found in the left lower quadrant and there was a palpable fecalith in the appendix. An appendectomy was performed. By the time the operation was concluded, the patient had been weaned off vasopressors, his lactate had improved to 2.2 mmol/L, and his creatinine had improved to 2.5 mg/dL. He had an uncomplicated post-operative course: his nasogastric tube was removed on post-operative day 4 and he was discharged home on post-operative day 6.

## Discussion and conclusions

The true prevalence of malrotation in both the pediatric and adult population is unknown due to the unclear number of asymptomatic patients. Adult presentation is rare and estimated to account for only 0.2–0.5% of cases [5]. The current literature suggests that presentation varies by age [4, 6, 7]. Malrotation in adults tends to either be found incidentally when a patient undergoes imaging or requires a laparotomy, or present as chronic, non-specific gastrointestinal symptoms such as intermittent crampy abdominal pain, bloating, weight loss, and nausea/vomiting. Right paraduodenal hernias are caused by abnormal rotation of the midgut during embryogenesis, secondary to failure of the pre-arterial limb to complete the 270º counterclockwise rotation [8, 9]. We present a rare case of a healthy 84-year-old with no previous gastrointestinal symptoms who was only diagnosed with malrotation when he presented acutely with bowel ischemia secondary to a paraduodenal hernia.

There are several findings on CT that are individually non-specific but, when taken together, are highly suggestive of intestinal malrotation. Firstly, the relationship of the SMA and SMV is abnormal. The SMA is normally to the left of the SMV. This relationship was found in 79% of normal individuals but only 29% of individuals with malrotation [10]. Secondly, the location of the third portion of the duodenum (D3) is abnormal. D3 should be retroperitoneal, coursing horizontally from right-to-left posteroinferior to the superior mesenteric artery and eventually terminating at the duodenojejunal flexure in the left upper quadrant. This course was identified in 100% of normal individuals but only 2.5% of individuals with malrotation [10] Thirdly, the location of the proximal small bowel is atypical. After the duodenal sweep, the jejunum is normally in the left mid-abdomen coursing toward the ileum in the right lower quadrant. Yet, 91% of patients with malrotation were found to have proximal small bowel predominantly in the right hemiabdomen [10]. Our patient demonstrated all three of these findings, thus there was a high radiological suspicion for malrotation.

There is a notable lack of quality data on screening for malrotation in asymptomatic patients. The current evidence is focused on patient populations that have a higher prevalence of malrotation including those with congenital heart disease such as heterotaxy syndrome [11] and abdominal wall defects [12]. An APSA systematic review on screening concluded that there is minimal Level 4 evidence to support screening in these high-risk groups, resulting in a Grade D recommendation [4]. This recommendation was based on several studies noting a poor distinction in what constituted normal anatomy in upper gastrointestinal contrast studies (UGI). Similarly, using an UGI for screening in the general population is not well established and has been associated with a 15% false positive [13] and 6% false negative rate [14]. Higher quality evidence is needed to produce a recommendation for screening in asymptomatic children and adults.

Moreover, there are no clear guidelines on the management of older asymptomatic patients with malrotation as the literature regarding the benefit of surgical intervention is varied. The traditional rationale to not offer surgery to older asymptomatic children and adults is based on the reported declining risk of presenting with volvulus with advancing age [13]. A systematic review comparing observation versus prophylactic Ladd’s procedure in patients with heterotaxy syndrome and asymptomatic malrotation found a 14% rate of post-operative complications in those who underwent a Ladd’s, including a 3% rate of peri-operative mortality, 10% rate of small bowel obstruction, and 21% long-term mortality rate (mostly due to heart failure) [15]. The authors concluded that they could not ascertain whether the risk of death from a prophylactic Ladd’s or the risk of missed midgut volvulus in patients who are observed was greater. Proponents of a prophylactic Ladd’s procedure to reduce future complications cite the lower peri-operative risks associated with an elective surgery. A retrospective study of 33 patients, ranging in age between 11 and 83 years, revealed that post-operative complications occurred significantly more frequently in patients operated on emergently versus electively (60 vs 22%) [16].

With our patient, we opted to perform an open Ladd’s procedure due to the acuity of his presentation, the extremely dilated stomach and duodenum, and the questionable etiology of his bowel ischemia given how rarely malrotation is diagnosed in his age group. The open approach is preferred in patients with confirmed or suspected midgut volvulus [17]. However, a Ladd’s procedure is often performed laparoscopically in the absence of bowel ischemia. There is evidence of an increased risk of volvulus recurrence with the laparoscopic approach in children due to the decreased formation of post-operative adhesions [17]. It is difficult to know whether malrotation caused or contributed to our patient’s bowel ischemia, or was an incidental finding in the setting of a paraduodenal hernia.

In conclusion, bowel ischemia secondary to a paraduodenal hernia in the setting of malrotation in a previously asymptomatic older adult is rarely encountered. While guidelines for screening and intervention in asymptomatic patients remain unclear, it is critical to keep malrotation on the differential in acutely ill patients with concerning presentations across pediatric and adult age groups. The prompt recognition and appropriate management of this potentially devastating condition will ultimately enhance patient outcomes.

### Patient perspective

The patient was surprised to learn about what had caused his acute presentation to the hospital, especially as he had been very healthy his entire life. The patient’s family was curious whether there was a genetic component to malrotation, and they were counseled that it is not typically thought of as a hereditary disease. The patient was thankful that no bowel had to be resected, and that the problem was solved during a single operation. He had been counselled that he may become quite ill post-operatively given his multi-organ dysfunction on presentation. However, on post-operative day #1 he already felt very well, and overall felt like his recovery was quite smooth.

## Supplementary Information

**Additional file 1: Figure S1.** Diagram of intra-operative anatomy. The duodenojejunal flexure was to the right of the spinal column and a right paraduodenal space was noted. Several congenital bands were found at the level of the duodenum and proximal jejunum. The cecum was in the left lower quadrant.

## Data Availability

Data sharing is not applicable to this article as no datasets were generated or analysed during the current study.

## Abbreviations

APSA: American Pediatric Surgical Association
CT: Computerized tomography
D3: Third portion of the duodenum
SMA: Superior mesenteric artery
SMV: Superior mesenteric vein
UGI: Upper gastrointestinal contrast studies
