# Supplementary material for: A problem at any age: a case report of congenital malrotation with bowel ischemia in an 84-year-old
Source: BMC Surg. 2022 Jan 29;22:35. doi: 10.1186/s12893-022-01482-6 (PMC8800360; doi:10.1186/s12893-022-01482-6)

**Additional file 1: Figure S1. Diagram of intra-operative anatomy**. The duodenojejunal flexure was to the right of the spinal column and a right paraduodenal space was noted. Several congenital bands were found at the level of the duodenum and proximal jejunum. The cecum was in the left lower quadrant.

**Additional file 1: Figure S1.**


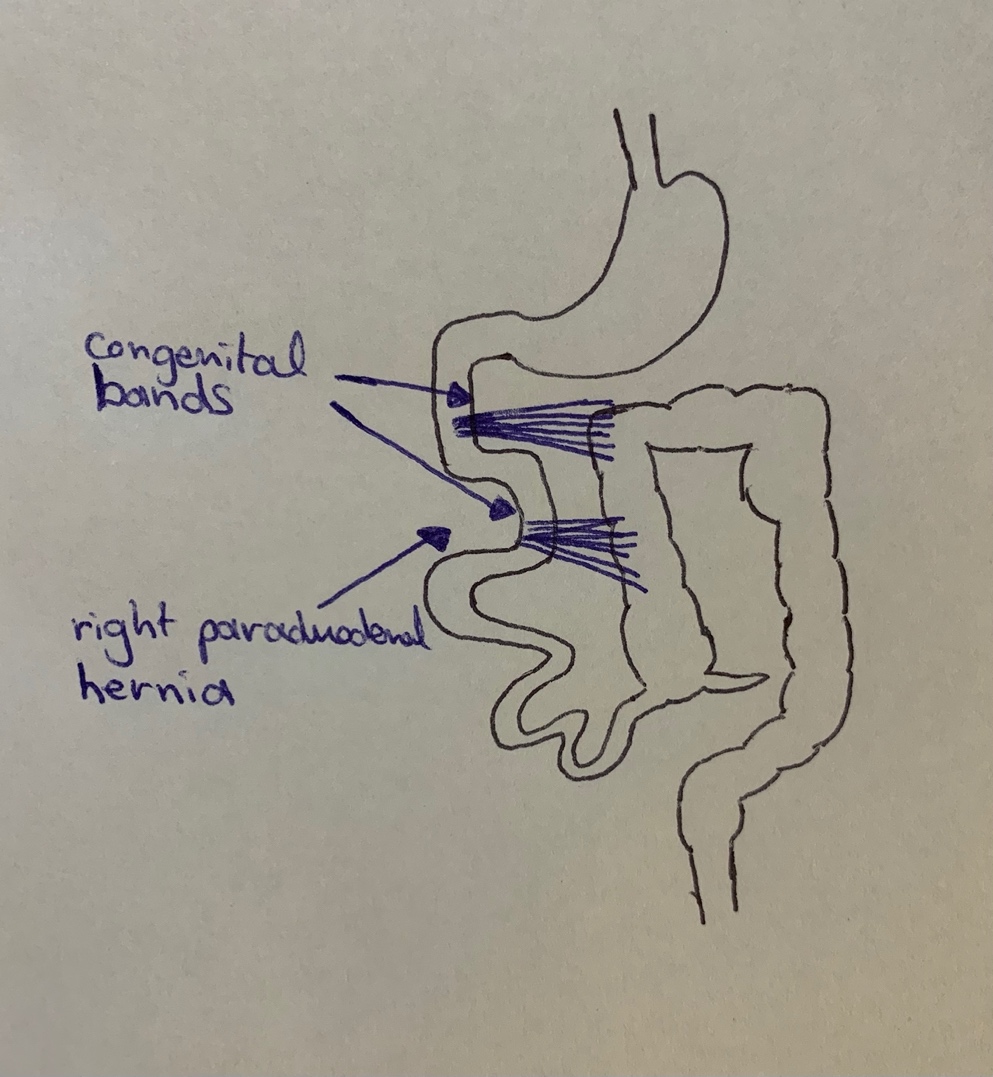

Supplement: Supplementary file 1 — Additional file 1: Figure S1. Diagram of intra-operative anatomy. The duodenojejunal flexure was to the right of the spinal column and a right paraduodenal space was noted. Several congenital bands were found at the level of the duodenum and proximal jejunum. The cecum was in the left lower quadrant. [file 12893_2022_1482_MOESM1_ESM.docx]
